# Comprehensive evaluation of lifespan‐extending molecules in *C. elegans*

**DOI:** 10.1111/acel.14424

**Published:** 2025-01-24

**Authors:** Grace B. Phelps, Jonas Morin, Carla Pinto, Lucas Schoenfeldt, Sebastien Guilmot, Alejandro Ocampo, Kevin Perez

**Affiliations:** ^1^ EPITERNA Epalinges Switzerland; ^2^ Department of Biomedical Sciences, Faculty of Biology and Medicine University of Lausanne Lausanne Switzerland

**Keywords:** aging, *C. elegans*, diet, drugs, lifespan, screening, worms

## Abstract

The nematode *C. elegans* has long served as a gold‐standard model organism in aging research, particularly since the discovery of long‐lived mutants in conserved aging pathways including *daf‐2* (IGF1) and *age‐1* (PI3K). Its short lifespan and small size make it highly suitable for high‐throughput experiments. While numerous molecules have been tested for their effects on *C. elegans* lifespan, consensus is still lacking regarding the most effective and reproducible compounds. Confounding effects, especially those related to drug‐bacteria interactions, remain a contentious issue in the literature. In this study, we evaluated 16 of the most frequently reported lifespan‐extending molecules in *C. elegans*, examining their effects on lifespan with two different diets (live and UV‐killed OP50). In addition, we assessed the compounds' impact on bacterial growth, their effects on various nematode strains, and the impact of the starting age of treatment. Our findings first confirmed robust lifespan extension by many, but not all, of the 16 tested compounds from the literature, and revealed that some of them could be combined to obtain additive effects. Additionally, we showed that some of these compounds also extend lifespan in the fly *D. melanogaster,* demonstrating a conserved effect across species. Finally, by expanding our screen to a broader pool of molecules, we identified novel lifespan‐extending compounds in *C. elegans*.

## Graphical abstract

We evaluated 16 of the most frequently reported lifespan‐extending molecules in *C. elegans*, examining their effects on lifespan with live and UV‐killed OP50. We assessed the compounds' impact on bacterial growth, and their effects on various nematode strains. We confirmed robust lifespan extension by many, but not all, of the 16 tested compounds, and revealed some potential for combinatorial additive effects. Lastly, by expanding our screen to a broader pool of molecules, we identified novel lifespan‐extending compounds in *C. elegans*.

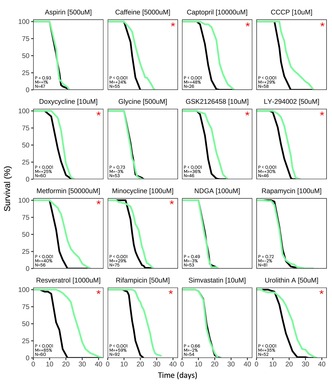

**Abbreviations**
ACE: angiotensin‐converting enzyme
CCCP: carbonyl cyanide m‐chlorophenyl hydrazone
CITP: *C. elegans* intervention testing program
Cox‐PH: Cox proportional hazards
FDR: false‐discovery rate
FUdR: 5‐Fluoro‐2 deoxyuridine
IGF1: insulin‐like growth factor 1
ITP: intervention testing program
mTOR: mammalian target of rapamycin
NDGA: Nordihydroguaiaretic acid
NGM: nematode growth medium
PI3K: Phosphoinositide 3‐kinase inhibitor
UV: ultraviolet

## 1 INTRODUCTION

The incidence of age‐related diseases has dramatically risen due to an increasingly aging population, posing a significant burden on society and health care systems. It is therefore essential to identify therapeutic strategies that can prevent the functional decline associated with aging, the occurrence of age‐related diseases and delay mortality in the elderly. The nematode *C. elegans* has a long history of being a gold standard model organism for aging research, notably with the identification of long‐lived mutants in conserved aging pathways including *daf‐2* (Kenyon et al., 1993) (IGF1) and *age‐1* (Friedman & Johnson, 1988) (PI3K). Its short lifespan and small size make it amenable for large‐scale experiments. Although many compounds have been tested for their effect on *C. elegans* lifespan, there is still a lack of consensus on which ones are the best and most reproducible (Bass et al., 2007; Wood et al., 2004). Confounding effects are also largely debated in the literature, notably concerning the interaction between the compound and the bacteria that are being fed to the worms (Beydoun et al., 2021; Cabreiro et al., 2013). Recent efforts like the *C. elegans* intervention testing program (CITP) (Lucanic et al., 2017), counterpart of the mouse intervention testing program (ITP) (Strong et al., 2022), have attempted to standardize testing of lifespan‐extending compounds in *C. elegans*, but so far with a relatively low number of compounds tested. Other approaches like the WormBot (Lee et al., 2023) may facilitate higher throughput experiments in worms.

In this work, we surveyed 16 of the most extensively reported lifespan‐extending compounds in *C. elegans*. We tested their effect on lifespan using two different types of food (live and UV‐killed *E. coli* OP50). Moreover, we assessed the effect of the compounds on the bacteria itself, as well as their effect on the lifespan of different nematode strains, and at multiple ages. In addition, we identified several new combinations of compounds with an additive effect on lifespan. Importantly, we also showed that some of these compounds extend lifespan in *D. melanogaster*, demonstrating a conserved effect across species. Lastly, we opened our screen to a larger pool of compounds and identified novel lifespan‐extending molecules in *C. elegans*.

## 2 RESULTS

### 2.1 Effect on lifespan of 16 of the most frequently reported lifespan‐extending compounds in *C. elegans* fed live OP50

We first sought to validate multiple lifespan‐extending compounds reported in the literature. For this, we surveyed DrugAge (Barardo et al., 2017), a database for lifespan extension data in different model organisms, and further performed a manual search for experiments performed at 20°C in solid media. This yielded an extensive list of putative lifespan‐extending compounds in the *C. elegans* strain N2. We specifically focused on a panel of 16 compounds: aspirin (Wan et al., 2013), the angiotensin‐converting enzyme (ACE) inhibitor captopril (Kumar et al., 2016), carbonyl cyanide m‐chlorophenyl hydrazone (CCCP) (Lemire et al., 2009), the dual mammalian target of rapamycin (mTOR)/ Phosphoinositide 3‐kinase inhibitor (*PI3K*) inhibitor GSK2126458, the PI3K inhibitor LY‐294002 (Calvert et al., 2016), metformin (Cabreiro et al., 2013), the antibiotics doxycycline (Bonuccelli et al., 2023), minocycline (Solis et al., 2018), and rifampicin (Golegaonkar et al., 2015), nordihydroguaiaretic acid (NDGA) (Banse et al., 2024), the mTOR inhibitor rapamycin (Robida‐Stubbs et al., 2012), the statin simvastatin (Jahn et al., 2020), and the natural compounds glycine (Liu et al., 2019), caffeine (Sutphin et al., 2012), resveratrol (Chen et al., 2013) and urolithin A (Ryu et al., 2016).

**FIGURE 1:**
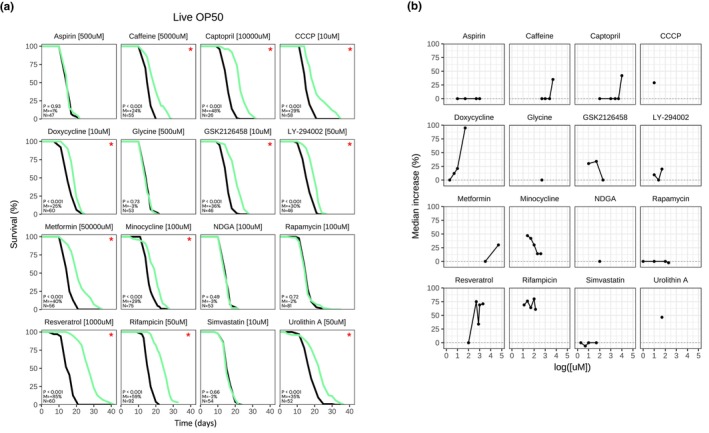
Effect on lifespan of 16 drugs in *C. elegans*, fed live OP50. (a) Survival curves for N2 treated with “Drug [Concentration]”, fed live OP50 at 20°C. P: *p*‐value in the logrank test. M: Median lifespan extension. N: Sample size of the treated group. “*”: *p* < 0.05. Treated group in green, control group in black. Time in days from hatching. Representative result. (b) Dose response for each drug. Median lifespan increase (*p* < 0.05) in *y*‐axis, log of concentration in *x*‐axis in μM. Each dot represents a drug‐concentration pair. Median extension = 0% if insignificant (*p* ≥ 0.05).

Since most of the compounds that we tested were dissolved in DMSO, we first tested the impact of DMSO concentration on *C. elegans* N2 lifespan with a live *E. coli* OP50 diet. Notably, increasing concentrations of DMSO up to 0.5% led to a slight, but significant, change in control median lifespan (15.2 vs. 15.8 days for H_2_O vs. 0.5% DMSO vehicle; Figure S1A,B), as previously reported (Wang et al., 2010). DMSO concentrations from 1% to 2% more dramatically extended N2 lifespan (median >20 days) and was toxic at ≥5% DMSO (Figure S1A,B). Subsequently, all lifespan experiments were carried out with up to 0.5% DMSO vehicle in both treated and control groups. We tested each compound for its ability to extend N2 survival when supplemented in the agar starting at the L4 stage under standard growth conditions with a live *E. coli* OP50 diet. Importantly, resveratrol (1 mM; ΔMed = 85%), rifampicin (50 μM; ΔMed = 59%), captopril (10 mM; ΔMed = 48%), metformin (50 mM; ΔMed = 40%), GSK2126458 (10 μM; ΔMed = 36%), urolithin A (50 μM; ΔMed = 35%), LY‐294002 (50 μM; ΔMed = 30%), CCCP (10 μM; ΔMed = 29%), minocycline (100 μM; ΔMed = 29%), doxycycline (10 μM; ΔMed = 25%), and caffeine (5 mM; ΔMed = 24%) significantly extended N2 lifespan at concentrations previously reported in the literature, further validating previous findings (Figure 1a).

We further tested several of these compounds at multiple concentrations to determine if there was a dose–response effect. Indeed, caffeine and captopril did not extend lifespan at lower concentrations (Figure 1b) and minocycline had a reduced effect at higher concentrations (Figure 1b). On the other hand, doxycycline extended lifespan at relatively low concentrations, up to and including 50 μM (Figure 1b). GSK2126458 best extended N2 lifespan when tested at 10 μM, still retained a positive effect when tested at 50 μM, and was no longer able to extend lifespan at 200 μM (Figure 1b). Resveratrol extended lifespan at high concentrations (≥500 μM) up to 2 mM, and rifampicin extended lifespan at all tested concentrations (Figure 1b). Surprisingly, 5/16 compounds in our literature‐curated panel did not extend lifespan in our system. Namely, neither aspirin (500 μM), glycine (500 μM), NDGA (100 μM), rapamycin (100 μM), nor simvastatin (10 μM) significantly impacted N2 survival (Figure 1a). Moreover, testing several of these compounds at multiple concentrations did not yield any positive results (Figure 1b). Overall, these data further support resveratrol, rifampicin, urolithin A, captopril, caffeine, metformin, CCCP, minocycline, doxycycline, GSK2126458, and LY‐294002 as lifespan‐extending compounds in *C. elegans*. Conversely, our data suggest that aspirin, glycine, NDGA, rapamycin, and simvastatin do not extend the lifespan of *C. elegans* fed live OP50.

### 2.2 Resveratrol, caffeine, metformin, doxycycline, GSK2126458, and LY‐294002 extend *C. elegans* lifespan fed a UV‐killed OP50 diet

**FIGURE 2:**
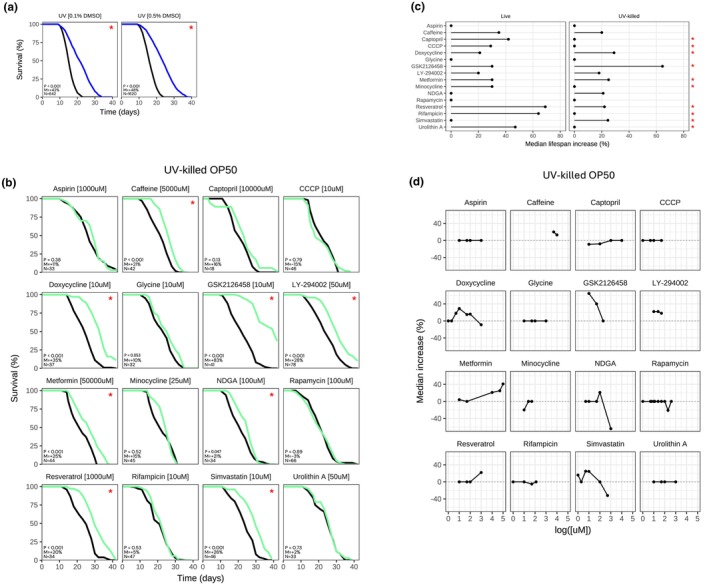
Resveratrol, caffeine, metformin, doxycycline, GSK2126458, and LY‐294002 still extend *C. elegans* lifespan, fed a UV‐killed OP50 diet. (a) Effect of UV‐killed OP50 on wild‐type N2 lifespan at 20°C, with exposure to 0.1% or 0.5% DMSO vehicle. (b) Survival curves for N2 treated with “Drug [Concentration]”, fed UV‐killed OP50. P: *p*‐value in the logrank test. M: Median lifespan extension. N: Sample size of the treated group. “*”: *p* < 0.05. Treated group in green, control group in black. Time in days from hatching. Representative result. (**c**) Summary of the effect of 16 drugs in live and UV‐killed OP50 on N2 median lifespan, at the best concentration tested. Median extension = 0% if insignificant (*p* ≥ 0.05). “*”: *p* < 0.05 for the interaction term in the survival model “lifespan ~ drug + diet + drug:Diet” (d) Dose response for each drug. Median lifespan increase (*p* < 0.05) in *y*‐axis, log of concentration in *x*‐axis in μM. Each dot represents a drug‐concentration pair. Median extension = 0% if insignificant (*p* ≥ 0.05).

Previous studies have found that the standard *C. elegans* diet, live *E. coli* strain OP50, could have a deleterious effect on lifespan due to the pathogenicity of the bacteria, potentially by causing pharyngeal swelling (Zhao et al., 2017). Additionally, it has been reported that some compounds may interact with live bacteria, which could be responsible for their effect on lifespan (Cabreiro et al., 2013). To avoid these confounding effects, we decided to additionally perform lifespan experiments on N2 fed UV‐killed OP50. First, we determined the impact of UV‐killed OP50 on N2 lifespan. As previously observed (Gems & Riddle, 2000), *C. elegans* on a UV‐killed OP50 diet lived significantly longer than those on a live OP50 diet (ΔMed = 42%–48%), supporting the argument that live OP50 is pathogenic to *C. elegans* (Figure 2a; Figure S1A,B).

Here again, we first tested the impact of DMSO concentration on N2 lifespan fed with UV‐killed OP50. Notably, we found that the percentage of DMSO in the agar impacted *C. elegans* lifespan. Namely, DMSO concentrations up to 2% increasingly extended N2 lifespan and was toxic at ≥5% DMSO (Figure S1A,B). For this reason, we sought to keep the concentration to ≤0.2% and only up to 0.5% in necessary cases. Next, we tested our literature‐curated panel of 16 compounds at multiple concentrations in *C. elegans* fed UV‐killed OP50 (Figure 2b–d). Of the 11 compounds that extended survival with live OP50, 6 compounds still reproducibly extended lifespan when fed UV‐killed OP50, including resveratrol (1 mM; ΔMed = 20%), metformin (50 mM; ΔMed = 25%), GSK2126458 (10 μM; ΔMed = 83%), LY‐294002 (50 μM; ΔMed = 28%), doxycycline (10 μM; ΔMed = 35%), and caffeine (5 mM; ΔMed = 21%). Interestingly, resveratrol and metformin extended lifespan to a lesser effect when fed UV‐killed OP50 than live OP50. Conversely, GSK2126458, and to a lesser extent doxycycline, had a greater impact on median lifespan when fed UV‐killed OP50. Overall, these six compounds were able to extend *C. elegans* lifespan with both live and UV‐killed OP50. Separately, of the 5 compounds that did not extend N2 lifespan when fed live OP50, 3 of them continued to fail to extend lifespan when tested at ≥4 concentrations (aspirin, glycine, and rapamycin) when fed UV‐killed OP50 (Figure 2d). Conversely, the other two compounds NDGA and simvastatin extended lifespan with UV‐killed OP50 at one concentration at least (Figure 2d), suggesting that their effect may only be seen in that context. We further tested in an interaction model whether some of the drugs extend lifespan depending on the bacteria being fed to the worm (live or UV‐killed OP50). In this model, for 10 of the 13 compounds that had a positive effect when fed either live or UV‐killed food, the effect was dependent on the bacteria being fed to the worms (*p* < 0.05 for the interaction term) (Figure 2c). Altogether, these results demonstrate that a live or UV‐killed OP50 diet can impact the potential lifespan‐extending effect of a compound in *C. elegans*.

### 2.3 Direct effect of the compounds on OP50 viability

Of the 5 compounds that extended lifespan with live OP50 but not with UV‐killed OP50, 3 of them (rifampicin, minocycline, and CCCP) are known to decrease live *E. coli* viability. Given our finding that UV‐killed OP50 increases N2 survival, likely due, at least in part, to pathogenicity of live *E. coli*, this raises the possibility that these three compounds increased lifespan when fed live OP50 by directly killing the bacteria rather than influencing the worms itself. To confirm this hypothesis, we sought to directly assess the effect of positive hits from the live OP50 screen on the bacteria alone. To accomplish this, we incubated each compound with seven 1:10 serial dilutions of live OP50 in a 96‐well plate containing LB media overnight at 37°C and observed OP50 growth the next morning.

Out of the 11 compounds that extended lifespan with live OP50 diet, 7 directly negatively affected OP50. We first confirmed that rifampicin, minocycline, and CCCP did indeed decrease live OP50 viability, however to different degrees—minocycline completely killed OP50 regardless of concentration, rifampicin only after the first 1:10 dilution, and CCCP only decreased OP50 thickness at each dilution, instead of completely killing it (Table 1, Figure S2). Notably, doxycycline entirely killed OP50 regardless of OP50 concentration, similarly to minocycline, which is unsurprising given the fact that they are known antibiotics. More surprisingly, we found that captopril, metformin, and to a lesser extent caffeine, also decreased OP50 viability (Table 1, Figure S2).

**TABLE 1:** Direct effect of the drug on OP50 viability. Direct effect of the drug‐concentration tested on the OP50 alone. Summary of the effect of 16 drugs in live and UV‐killed OP50 on N2 median lifespan at 20°C. The impact of the drug on OP50 was determined by the percentage of wells with viable OP50 at the multiple OP50 dilutions tested (7 1:10 serial dilutions starting from 0.3 mg/mL OP50) and the quality of the OP50 pellets. Quality of OP50 pellets was determined by visual assessment (Figure S2).

Drug	Conc. (μM)	Δmedian lifespan with live OP50	Δmedian lifespan with UV‐killed OP50	Drug negatively affects OP50?	% Wells with OP50	Overall quality of OP50 pellet
Resveratrol	1000	82%	20%	No	100%	Normal
Rifampicin	10	69%	0%	Yes	14%	Small
Urolithin A	50	56%	0%	No	100%	Normal
Captopril	10000	53%	0%	Yes	14%	Sparse
CCCP	10	45%	0%	Yes	100%	Sparse
Caffeine	5000	44%	21%	Yes	100%	Sparse
Metformin	50000	37%	25%	Yes	29%	Sparse
Minocycline	25	37%	0%	Yes	0%	N/A
LY‐294002	50	24%	28%	No	100%	Normal
Doxycycline	10	23%	35%	Yes	0%	N/A
Simvastatin	10	0%	26%	No	100%	Normal

Importantly, resveratrol, urolithin A and LY‐294002 did not affect the OP50, suggesting that their lifespan‐extending effect cannot be attributed to decreasing bacteria viability. Additionally, resveratrol and LY‐294002 also extended lifespan of worms fed with UV‐killed OP50. On the other hand, rifampicin, captopril, CCCP, minocycline extended lifespan of *C. elegans* only when fed live OP50, and directly reduced OP50 viability, suggesting that their lifespan‐extending effect may be due to a negative impact of the compound on OP50. Caffeine, metformin, and doxycycline affected *E. coli* viability, but also extended lifespan when fed UV‐killed OP50, therefore their positive effect on lifespan cannot be explained by their effect on the bacteria alone. Taken together, these results demonstrate that the effect of compounds on lifespan when fed live *E. coli* cannot be entirely explained by their effect on the bacteria.

### 2.4 Combination studies identify beneficial drug pairs

**FIGURE 3:**
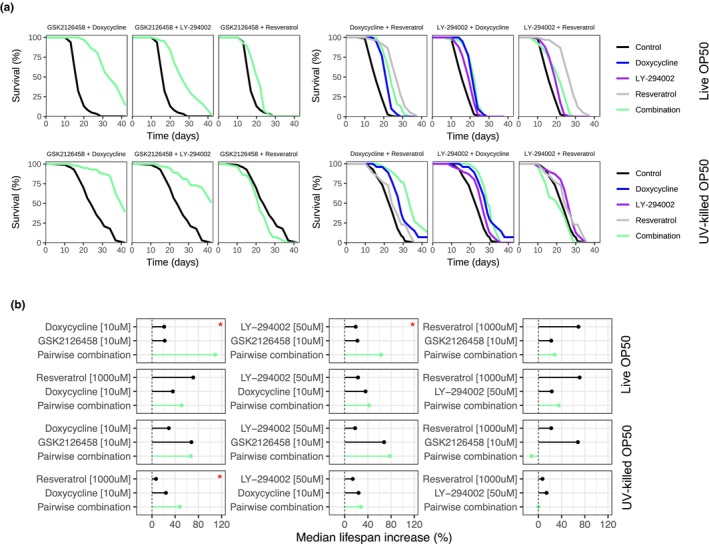
Combination studies identify beneficial drug pairs. (a) Survival curves for pairwise combinations of drugs tested on N2 lifespan at 20°C when fed live OP50. Control in black, single drugs in blue, gray, purple, pairwise combination in light green. “*”: *p* < 0.05 for comparison of combination survival to all the single‐drug results. (b) Summary effect of the single drugs and combinations on N2 lifespan from the survival curves. Median extension of the single compounds for comparison to the GSK2126458 combinations is a representative result. Median extension = 0% if insignificant (*p* ≥ 0.05). “*” indicates combination result is significant compared to each single drug alone.

After confirming which of the 16 compounds robustly extended *C. elegans* lifespan, we next sought to determine if additive effects could be observed by combining some of the positive hits from our initial screen. Indeed, combinatorial interventions hold the potential to increase effect sizes, by targeting multiple aging pathways at the same time. More precisely, we tested pairwise combinations of four of our best hits, namely doxycycline (10 μM), LY‐294002 (50 μM), GSK2126458 (10 μM), and resveratrol (1 mM); both with live and UV‐killed OP50 diets. We found that many combinations did not yield any significant increase in lifespan extension, compared to the single compounds alone (13 out of 16 combinations, Figure 3a,b). Surprisingly, some combinations even canceled the positive effect of the single compounds alone. Combinations of resveratrol with GSK2126458 or LY‐294002 were largely inferior to resveratrol alone when fed live OP50. With a UV‐killed diet, the combination of resveratrol with GSK2126458 was inferior to both resveratrol and GSK2126458 alone, even reaching slight toxicity. On the other hand, combination of GSK2126458 with doxycycline or LY‐294002 dramatically increased the effect of each single drug alone with a live OP50 diet (*p* < 0.001 or *p* = 0.01, respectively, for the combination compared to best single compound). With a UV‐killed diet, combining doxycycline and resveratrol also had an increased effect compared to single drugs alone (*p* = 0.0413, for the combination compared to best single compound, Figure 3a,b).

Next, we tested pairwise combinations of two other drug triplets: minocycline (100 μM), caffeine (5 mM), and rifampicin (50 μM), with a live OP50 diet; and LY‐294002 (50 μM), caffeine (5 mM), and metformin (50 mM), with a UV‐killed OP50 diet. 5 out of 6 combinations did not yield any significant beneficial effect, compared to single molecules alone. However, the combination of rifampicin and caffeine had an additive impact on median lifespan (*p* < 0.001, for the combination compared to best single compound, Figure S3A,B). Lastly, we performed a 3 × 3 dose escalation study for the combination of doxycycline and resveratrol in UV‐killed conditions (Figure S3C). Interestingly, 3 doses (2 mM + 5 μM, 0.5 mM + 10 μM, 2 mM + 20 μM) seem to reach comparable or greater effectiveness than the original selected dose (1 mM + 10 μM). Additionally, one dose (0.5 mM + 20 μM) did not yield to any positive effect. Taken altogether, these results demonstrate a relatively low hit rate for combination experiments, but suggest a possible benefit in the combination of GSK2126458 with LY‐294002 or doxycycline and argue against combinations of resveratrol with LY‐294002 or GSK2126458.

### 2.5 Impact of resveratrol and LY‐294002 on lifespan of other Caenorhabditis species, strains and mutants

**FIGURE 4:**
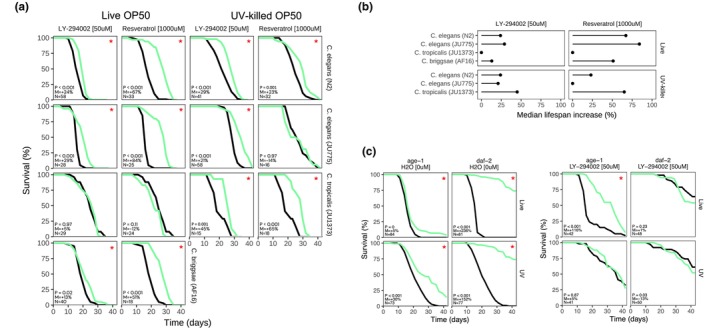
Impact of resveratrol and LY‐294002 on lifespan of other Caenorhabditis nematodes. (a) Survival curves for *C. elegans* (N2 or JU775), *C. tropicalis* (JU1373), and *C. briggsae* (AF16) treated with LY‐294002 (50 μM) or resveratrol (1000 μM), fed either live or UV‐killed OP50 at 20°C. P: *p*‐value in the logrank test. M: Median lifespan extension. N: Sample size of the treated group. “*”: *p* < 0.05. Treated group in green, control group in black. Time in days from hatching. (b) Summary effect of LY‐294002 (50 μM) or resveratrol (1000 μM) on Caenorhabditis change in median survival relative to vehicle control. Median extension = 0% if insignificant (*p* ≥ 0.05). (c) Survival curves for *C. elegans daf‐2* (*e1370*) and *age‐1* (*hx546*) mutants compared to N2 (left). Survival curves for *C. elegans daf‐2* and *age‐1* mutants, treated with LY‐294002 compared to vehicle controls.

As the effect of molecules in *C. elegans* has been reported to be strain‐dependent (Lucanic et al., 2017), we sought to test the effect of two of our best compounds in multiple nematode species and strains. For this reason, we tested the effect of LY‐294002 and resveratrol in *C. elegans* (N2, JU775), as well as *C. tropicalis* (JU1373), and *C. briggsae* (AF16) with both live and UV‐killed OP50 diets. Interestingly, LY‐294002 (50 μM) significantly extended JU775 lifespan when fed live OP50 (ΔMed = 29%) and UV‐killed OP50 (ΔMed = 21%). Resveratrol (1 mM) extended JU775 lifespan with live OP50 (ΔMed = 84%) but did not impact lifespan when fed UV‐killed OP50 (Figure 4a,b). We found that LY‐294002 (50 μM) and resveratrol (1 mM) did not impact JU1373 when fed live OP50 (Figure 4a,b), but both compounds significantly increased JU1373 median lifespan when fed UV‐killed OP50 (ΔMed = 45% or 65%, respectively; Figure 4a,b). Interestingly, we found that AF16 did not tolerate the UV‐killed OP50 diet, and largely fled the wells containing this diet (data not shown). Additionally, both LY‐294002 (50 μM) and resveratrol (1 mM) significantly extended AF16 lifespan (ΔMed = 13% or 51%, respectively; Figure 4a,b). Overall, the lifespan‐extending effect of resveratrol, and more strongly LY‐294002, was conserved across most of the conditions tested in these different strains and diets.

Since LY‐294002 is known to target the PI3K pathway, we next tested whether its lifespan‐extending effect would be abrogated in an *age‐1* (*hx546*) (PI3K) or *daf‐2* (*e1370*) (IGF1) mutant background, reinforcing its mechanism of action (MoA) in *C. elegans*. As expected, *daf‐2* extended lifespan by 236% when fed live OP50 and 152% when fed UV‐killed OP50, while *age‐1* also extended lifespan by only 5% when fed live food and 30% when fed UV‐killed food, compared to N2 controls. However, LY‐294002, which extends N2 lifespan, did not extend *age‐1 or daf‐2* lifespan compared to vehicle controls, except for *age‐1* with a live OP50 diet, suggesting that LY‐294002 may indeed also act through the PI3K or IGF1 pathway in worms (Figure 4c).

### 2.6 Doxycycline, caffeine, and GSK2126458 extend lifespan when administered at day 1 of adulthood

When evaluating the effect of a compounds on the lifespan of organisms, the starting time of treatment is critical. While some compounds in the literature have been shown to be able to extend lifespan only when given during development, others are able to retain their effect even when given at a later stage in life (Aiello et al., 2022; Bennett et al., 2023). In this study, we exposed worms to the compounds for their entire adult lifespan starting at the late L4 stage, the final larval stage. The reason for this protocol is that this also coincides with the strict timing of FUdR, a chemical that suppresses progeny formation, which is used in most lifespan experiments. Thus, we wondered if by delaying compound treatment by just 1 day, allowing worms to fully reach adulthood, we would see a difference in the effect of the compounds on lifespan. Thus, we transferred day 1 adult N2 *C. elegans* to experimental plates containing a subset of our best positive compounds, fed a diet of live or UV‐killed OP50.

**FIGURE 5:**
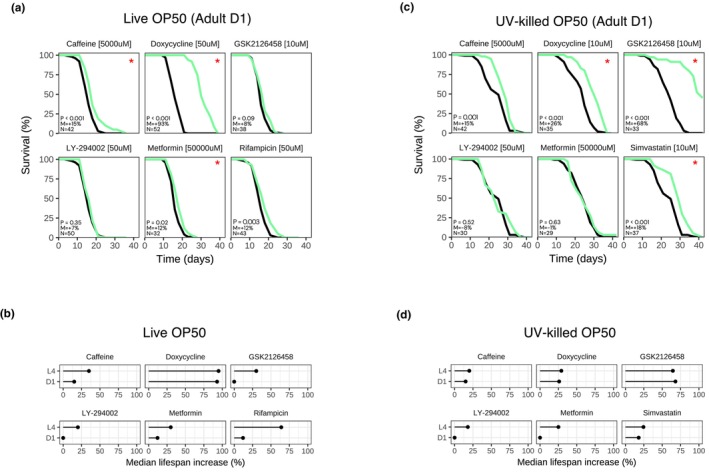
Doxycycline and caffeine extend lifespan when administered at day 1 of adulthood with both a live and UV‐killed OP50 diet. (a) Survival curves for N2 treated with “Drug [Concentration]” starting at day 1 of adulthood at 20°C, fed live OP50. P: *p*‐value in the logrank test. M: Median lifespan extension. N: Sample size of the treated group. “*”: *p* < 0.05. Treated group in green, control group in black. Time in days from hatching. (b) Summary effect of each drug on median lifespan extension when administered at late L4 versus day 1 of adulthood, fed live OP50. Median extension = 0% if insignificant (*p* ≥ 0.05). (c) Survival curves for N2 treated with “Drug [Concentration]” starting at day 1 of adulthood, fed UV‐killed OP50. P: *p*‐value in the CoxPH model. M: Median lifespan extension. N: Sample size of the treated group. “*”: *p* < 0.05. Treated group in green, control group in black. Time in days from hatching. (d) Summary effect of each drug on median lifespan extension when administered at late L4 versus day 1 of adulthood, fed UV‐killed OP50. Median extension = 0% if insignificant (*p* ≥ 0.05).

Notably, we found that doxycycline extended *C. elegans* lifespan when administered at L4 and day 1 adult stages, regardless of diet and concentration tested (Figure 5a–d, Table S1). Moreover, caffeine (5 mM), metformin (50 mM), resveratrol (800 μM), rifampicin (50 μM), and urolithin A (50 μM), still significantly extended N2 survival when administered at day 1 of adulthood and fed live OP50, however median extension was less than what we typically observed when administered at L4 (Figure 5a,b, Table S1). Interestingly, LY‐294002 (50 μM) and GSK2126458 (10 μM), did not extend N2 lifespan when administered at day 1 and fed live OP50 (Figure 5a,b). When fed UV‐killed OP50, caffeine (5 mM) and simvastatin (10 μM) still extended lifespan when administered at day 1 of adulthood, with only a slight decrease in median extension compared to L4 (Figure 5c,d). Metformin was no longer able to extend lifespan when fed UV‐killed OP50 at day 1 (Figure 5c,d). Importantly, GSK2126458 (10 μM) greatly extended lifespan when administered at day 1 of adulthood when fed UV‐killed OP50 (ΔMed = 68%), comparable to its effect at the L4 stage, while LY‐294002 (50 μM) was also not able to extend lifespan at this stage (Figure 5c,d). Overall, most of the compounds evaluated extend lifespan when administered at day 1 of adulthood, although sometimes at lower potency when tested at the same concentration as the L4 experiments.

### 2.7 Effect of compounds on the size and shape of *C. elegans*

Next, we tested whether the selected compounds had any effect on the size and shape of N2 *C. elegans* when treated starting at the late L4 stage. Towards this goal, we assessed the length, width, and area of worms treated with selected compounds, compared to controls, using the WormLab software (MBF Bioscience) (Table S2). First, we observed that worms fed UV‐killed OP50 were significantly bigger than worms fed live OP50, both in length (*p* < 0.001) and area (*p* = 0.01). Next, we observed that most compounds did not significantly impact the shape of the worm. Yet, resveratrol treated worms with live OP50 were bigger, both in width (*p* < 0.001) and area (*p* = 0.001) than untreated controls, and LY‐294002 treated worms were smaller, in length (*p* = 0.02). For worms fed UV‐killed OP50, GSK2126458 treated worms were smaller in length, width, and area than untreated controls (*p* < 0.001 for all) and LY‐294002 treated worms were smaller in length (*p* < 0.001) and area (*p* = 0.03) than controls. Overall, most of the lifespan‐extending compounds tested did not affect the worms' size and shape, but it is interesting to note that the ones that did, did so in opposite directions.

### 2.8 Doxycycline, metformin, rifampicin, and LY‐294002 extend D. Melanogaster lifespan

**FIGURE 6:**
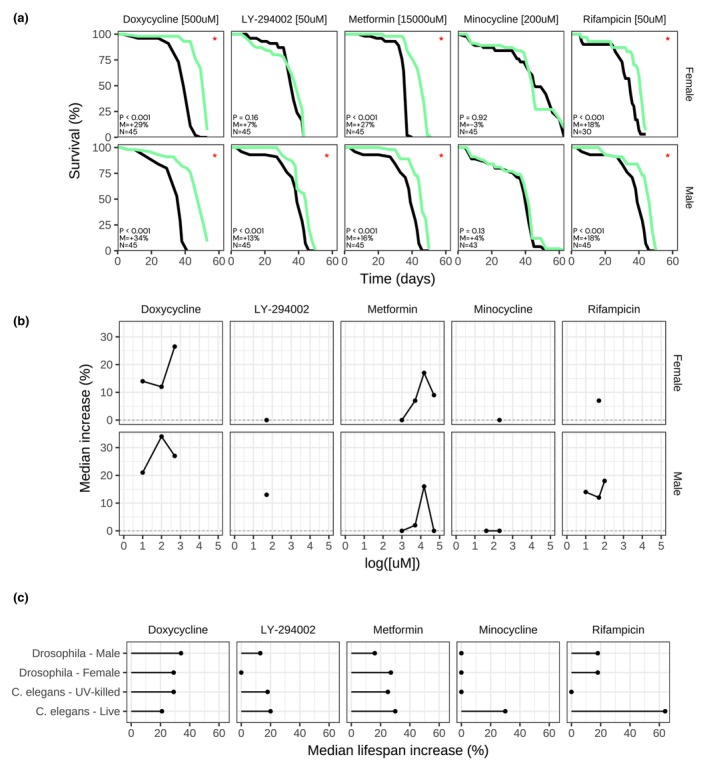
Doxycycline, metformin, rifampicin, and LY‐294002 extend *D. melanogaster* lifespan. (a) Survival curves for Canton S male or female *Drosophila melanogaster* at 25°C treated with “Drug [Concentration]”. P: *p*‐value in the logrank test. M: Median lifespan extension. N: Sample size of the treated group. “*”: *p* < 0.05. Treated group in green, control group in black. Representative result. (b) Dose response for each drug. Median lifespan increase (*p* < 0.05) in *y*‐axis, log of concentration in *x*‐axis in μM. Median extension = 0% if insignificant (*p* ≥ 0.05). (c) Summary effect of each drug on median lifespan extension for male or female *Drosophila* compared to N2 *C. elegans* fed either live or UV‐killed OP50. Median extension = 0% if insignificant (*p* ≥ 0.05).

We next asked whether the lifespan‐extending effect we observed in *C. elegans* could be conserved across species. For this reason, we tested a subset of the *C. elegans* positive molecules in a larger model organism, the fly *D. melanogaster*. Towards this goal, we incorporated doxycycline, metformin, rifampicin, LY‐294002, or minocycline into the fly diet, and measured their impact on *D. melanogaster* lifespan. Interestingly, we found that doxycycline (500 μM) significantly extended both male and female fly lifespan (ΔMed = 34% and 29%, respectively), and extended lifespan at lower concentrations (Figure 6). Additionally, metformin (15 mM) increased male and female lifespan (Δmed = 16% and 27%, respectively; Figure 6). Similarly, rifampicin (50 μM) increased male and female lifespan (ΔMed = 18% and 18%, respectively; Figure 6). LY‐294002 (50 μM) led to lifespan extension in male (ΔMed = 13%) but not female flies (Figure 6). Conversely, minocycline (200 μM) did not extend neither male nor female lifespan. Out of the 5 compounds that we tested 4 had a conserved effect on *D. melanogaster* lifespan extension, and one did not.

### 2.9 Large‐scale screening of putative lifespan‐extending compounds in *C. elegans* identifies miconazole, fenbendazole, tadalafil, lotilaner, and safinamide as novel candidates

**FIGURE 7:**
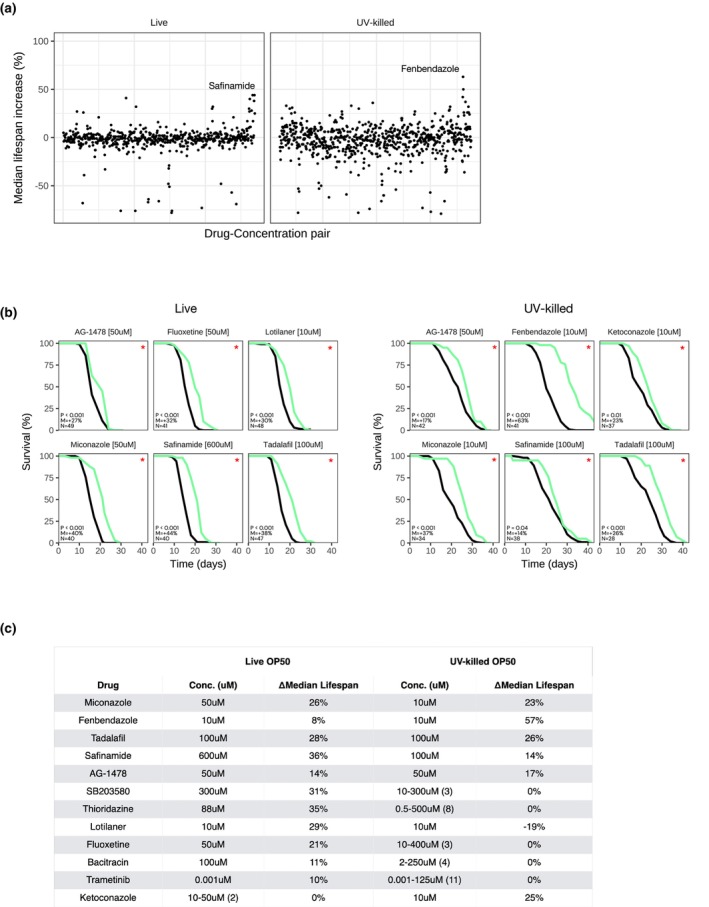
Large‐scale screening of putative lifespan‐extending compounds in *C. elegans*. (a) Dot‐plot of the impact of ≥200 compounds (≥2 concentrations tested) on N2 median lifespan at 20°C when fed live (left panel) or UV‐killed (right panel) OP50. Each dot represents and individual experiment. Median lifespan increase (%) in *y*‐axis and drugs in alphabetical order in *x*‐axis. (b) Survival curves for top lifespan‐extending drugs when N2 are fed live (left panel) or UV‐killed (right panel) OP50. (c) Summary of top survival results. Median extension = 0% if insignificant (*p* ≥ 0.05). The range of concentrations tested are listed with total number of concentrations tested in “()” for drugs that did not significantly change median lifespan.

We next expanded our study to screen more than 200 compounds for extension of N2 lifespan when administered at the late L4 stage and fed either live (195 compounds) or UV‐killed (228 compounds) OP50. Each compound was tested at a minimum of two different concentrations. Full survival results are included in Table S1. This small library contained compounds from different categories, such as antioxidants, anti‐inflammatories, antibiotics and anti‐fungals, PI3K inhibitors, anti‐diabetics, hormones, psychotropics, and anti‐hypertensives. Overall, of all the compounds and concentrations tested, 34/470 (7.2%) of experiments with live OP50 and 39/592 (6.7%) of experiments with UV‐killed OP50 yielded a significantly (*p* < 0.05) positive median lifespan extension (Figure 7a, Table S1). We also observed a low variability within our control conditions, when fed either live or UV‐killed OP50, suggesting high consistency (Figure S4).

We repeated the top hits from the screen and ultimately identified 11 compounds that reproducibly extended lifespan when fed live OP50 and 6 with UV‐killed OP50 (Figure 7b,c). 5 of these compounds extended lifespan when fed both live and UV‐killed OP50: the antifungal agent miconazole (ΔMed = 40% and 37%, respectively), the antiparasitic fenbendazole (ΔMed = 8% and 63%, respectively), the PDE5 inhibitor tadalafil (ΔMed = 38% and 26%, respectively), the MAO‐B inhibitor safinamide (ΔMed = 44% and 14%, respectively), and the tyrosine kinase inhibitor AG‐1478 (ΔMed = 27% and 17%, respectively; Figure 7b,c). Of these 5, only AG‐1478 had been previously tested and shown to extend lifespan in *C. elegans* (Ye et al., 2014).

Of the 11 compounds found to reproducibly extend lifespan in N2 when fed live OP50, 6 did not extend N2 lifespan when fed UV‐killed OP50 at ≥3 concentrations tested. Namely, the p38 MAPK inhibitor adezmapimod (SB203580) (ΔMed = 32%), the antibiotic bacitracin (ΔMed = 11%), the antidepressant fluoxetine (ΔMed = 32%), the anti‐parasitic lotilaner (ΔMed = 30%), and the antipsychotic thioridazine (ΔMed = 25%) extended lifespan only when fed live OP50 (Figure 7b). Of these compounds, thioridazine, fluoxetine, and bacitracin have previously been tested and shown to extend lifespan in *C. elegans* (Bonuccelli et al., 2023; Ye et al., 2014; Zhou et al., 2023), and adezmapimod has been shown to extend lifespan in *D. melanogaster* (Spindler et al., 2012). Additionally, we identified one compound that extended *C. elegans* lifespan when fed UV‐killed OP50 but not live OP50 when tested at ≥3 concentrations, the antifungal ketoconazole (ΔMed = 23%, Figure 7b).

We thus identify miconazole, fenbendazole, tadalafil, safinamide, adezmapimod (SB203580), lotilaner, and ketoconazole as novel lifespan‐extending compounds in *C. elegans*. We also validate the lifespan‐extending effects of thioridazine, fluoxetine, and bacitracin, dependent on OP50 diet.

## 3 DISCUSSION

In this study, we sought to validate a panel of 16 compounds reported to extend lifespan in *C. elegans*. We found that 11 compounds, including resveratrol, rifampicin, captopril, metformin, GSK2126458, and urolithin A, significantly extended lifespan when fed a live *E. coli* OP50 diet. We identified dose–response effects, particularly for caffeine and captopril, which required higher doses, while doxycycline and rifampicin were effective across a range of concentrations. We confirmed that a UV‐killed OP50 diet extends *C. elegans* lifespan, and revealed that only6 compounds (resveratrol, metformin, GSK2126458, LY‐294002, doxycycline, and caffeine) retained their lifespan‐extending effects with this diet. The reduced efficacy of rifampicin, captopril, CCCP, and minocycline with this diet suggests that their effect with live OP50 may be due to their antibiotic activity rather than a direct effect on *C. elegans*. This was further confirmed by assessing their impact on OP50 viability, where these compounds significantly reduced bacterial growth.

Combination studies identified beneficial drug pairs, notably rifampicin and caffeine, GSK2126458 and doxycycline, and GSK2126458 and LY‐294002, which together produced more than additive effects on lifespan extension. Conversely, combining resveratrol with LY‐294002 or GSK2126458 abolished their individual positive effects. In our study, combining two compounds with a positive effect on lifespan rarely led to an additive effect and sometimes canceled the positive effect observed in the single compounds. These results highlight the importance of combinatorial screenings for the identification of combinations of compounds with additive or synergistic effects of lifespan, and strongly suggest against the consumption of multiple supplements and drugs, which combined effects on health and lifespan have yet to be evaluated.

Moreover, we showed that resveratrol and LY‐294002 maintained their lifespan‐extending effects across various nematode species, strains, and diets, with some specific differences. However, the effect of LY‐294002 was canceled in a *daf‐2* mutant background, and partially in the *age‐1* mutant background, suggesting that it may extend lifespan through the IGF1/PI3K pathways. Furthermore, delayed compound administration, at day 1 of adulthood, generally retained efficacy, albeit sometimes with reduced potency. The effect of the compounds on worms' size and shape was also analyzed, and the compounds largely did not alter the worms shape, except GSK2126458 and LY‐294002 notably decreased worm size. Using *D. melanogaster*, we also show that the effect of some compounds on lifespan can be conserved across species, while others may be species dependent, increasing the potential translation of these discoveries to larger animals including companion animals and humans. Lastly, in a small proof‐of‐concept screening of ~200 compounds, we identified new compounds extending lifespan in *C. elegans*, including safinamide and fenbendazole. It is surprising to see that fenbendazole, an anthelmintic drug, extends *C. elegans* lifespan, and we can only suspect that this may be due to a hormetic effect. We also note that another anthelmintic drug, ivermectin, has also been shown to extend *C. elegans* lifespan (Zullo et al., 2019). Overall, several potential anti‐aging pathways are highlighted in this study, including PI3K (GSK2126458, LY‐294002), MAO (safinamide) and PDE5 (tadalafil) (Table S1).

In this study, we highlight the complex relationship between lifespan‐extending compounds and the *C. elegans* diet. In this regard, we find examples of compounds that extend lifespan with a live OP50 diet andtoxic to the bacteria; that extend lifespan with a live OP50 diet and not toxic to the bacteria; and that extend lifespan with a live OP50 diet, toxic to the bacteria, but also extending lifespan with UV‐killed OP50. Based on these results, it is difficult to conclude on the role of the interactions between compounds and the bacteria on *C. elegans* lifespan extension. Furthermore, the fact that some compounds extend lifespan with UV‐killed bacteria but not with live bacteria is also puzzling (NDGA, simvastatin), or that some may extend lifespan more in UV‐killed conditions than in live (GSK2126458). A possible interpretation would be that the drug is metabolized by live bacteria, rendering it ineffective. Further studies will be needed to increase our understanding of this important aspect. Until then, we recommend testing compounds with both live and UV‐killed OP50, and separately assessing the effect of the compounds on bacteria alone.

Previous studies have found that *C. elegans* lifespan, and the effect of putative lifespan‐extending molecules, can vary due to even subtle changes in the environment, compound suppliers, and experimental conditions (Lithgow et al., 2017; Stuhr & Curran, 2023; Urban et al., 2021). For example, rapamycin, a popular lifespan‐extending drug candidate, has been shown to extend lifespan across different species including *C. elegans* (Harrison et al., 2009; Robida‐Stubbs et al., 2012). However, other research groups, including the CITP, which specifically tests compounds in *C. elegans* in three different sites to thoroughly interrogate a candidate, have similarly not reproduced lifespan extension by rapamycin in *C. elegans* (Lucanic et al., 2017). Other groups have reported recently that the reason may be that rapamycin precipitates out of the agar, something we have also observed, depending on concentrations. Another drug, metformin, was shown to behave differently depending on the type of bacteria fed to the worm (Cabreiro et al., 2013). Specifically, at a similar dose of 50 mM, metformin was found to shorten *C. elegans* lifespan when fed UV‐killed bacteria, but with a slightly different UV‐killing protocol. Although we observe a slightly reduced effect of metformin in UV‐killed conditions, we do not observe this toxicity. Interestingly, resveratrol extended *C. elegans* lifespan under normal feeding conditions, while in mice it was shown that a high‐fat diet was necessary to observe its lifespan‐extending effect (Baur et al., 2006).

There are some limitations to this study. Since FUdR was used in all experiments to prevent progeny formation, we cannot exclude drug–drug interactions between the compounds tested and FUdR. Additionally, we did not assess whether the compounds tested were absorbed by the worms, or if the drug target (if known) was hit and to what degree, which might explain some negative results. Overall, our study demonstrates that while several compounds can extend lifespan in *C. elegans*, their efficacy is influenced by bacterial diet, dosage, strain, and starting age of the treatment. Our results underscore the importance of validating lifespan‐extending interventions under standardized conditions and exploring potential combinations for enhanced efficacy.

## 4 METHODS

### 4.1 *C. elegans* maintenance

Worms (N2, JU775, JU1373, AF16, TJ1052 [*age‐1* (*hx546*) II], CB1370 [*daf‐2* (*e1370*) III]) were obtained from the Caenorhabditis Genetics Center (CGC). Worms were maintained at 20°C and 80% humidity in standard solid NGM growth conditions (0.3% NaCl, 0.25% BactoPeptone, 2% agar, 1 mM MgSO₄, 1 mM CaCl₂, 5 μg/mL cholesterol, and 25 mM potassium phosphate buffer (pH 6.0]). Unless otherwise stated, worms were fed live OP50 obtained fresh from overnight inoculation of a single OP50 colony into LB at 37°C, concentrated to 30 mg/mL in ddH2O, and seeded on solid NGM agar plates. OP50 was obtained from the CGC.

### 4.2 Compounds

A complete list of the compounds tested, and catalog numbers is provided in Table S1. All compounds were dissolved in H_2_O or DMSO dependent on solubility.

### 4.3 Lifespan assay

Detailed and summarized data for all lifespan experiments carried out in this study can be found in Table S1. Worms were synchronized by 10‐min hypochlorite treatment (1% sodium hypochlorite, 800 mM sodium hydroxide, and water) and washed 4 times with M9 buffer to isolate embryos. Embryos were hatched overnight in M9 buffer on a rocker at 20°C (day = 0 on lifespan graphs). L1s were then transferred to NGM plates seeded with live OP50. Unless otherwise noted, worms were transferred to experimental plates at the late L4 stage (15 worms/well of a 24‐well plate; 3 wells per condition). For experiments that exposed worms to drug at day 1 of adulthood, late L4 worms were transferred to maintenance plates containing 150 μM 5‐Fluoro‐2′deoxyuridine (FUdR; calculated with respect to the volume of the agar to prevent progeny production). We chose a higher dose of FUdR to ensure consistent inhibition of progeny formation, but first confirmed that this dose does not influence *C. elegans* lifespan (Figure S1C). 24 h later, day 1 adult worms were transferred to experimental plates.

24‐well experimental plates were prepared by mixing drug or vehicle into NGM agar (1.5 mL/well) prior to solidification. Drug concentration was calculated with respect to the volume of the agar and treated and control wells contained an equal concentration of DMSO. Experimental plates were seeded with 30 μL of live or UV‐killed OP50 (60 mg/mL) mixed with 150 μM FUdR (calculated with respect to the volume of the agar). UV‐killed OP50 was prepared by exposing live OP50 diluted to 30 mg/mL to 254 nm high‐intensity UV light for 4 h on a UV transilluminator (TFS‐30 V) and subsequently concentrated to 60 mg/mL before seeding on experimental NGM plates. Of note, we have found that lower exposure times do not sufficiently inactivate the OP50. Experimental plates were wrapped in parafilm and kept at 20°C at 80% humidity for the duration of the experiment. Worm lifespan was assessed by movement over 48‐h periods using standard microscopy. All our lifespan experiments were done with *N* > 45 worms per condition, which gives us power to detect a 10% median lifespan extension with a confidence of 90% and *p*‐value of 0.05. All our positive results were repeated at least twice. When possible, the experiments with live and UV‐killed bacteria for the same drug were performed at the same time.

### 4.4 Survival analysis

Statistics for the survival analysis were obtained by fitting a proportional hazards regression model (Cox‐PH) on treated compared to control worms. Median extension, *p*‐value of the logrank test and sample size were reported.

### 4.5 Interaction between the drug and the bacteria

To test whether the effect of the drug was dependent on the bacteria, we ran a Coxph model with interaction term, like so: “survival ~ drug + diet + drug:diet”. We further used the *p*‐value of the interaction term to assess whether the drug extends lifespan depending on the OP50 diet.

### 4.6 Significance of combination assays

For combinatorial assays where we tested single compounds and combinations at the same time, the combination was deemed significant if it was significant compared to the best single compound. For combinatorial assays where we did not have the single compounds tested as part of the same experiment as the combination, we assessed significance using Fisher's *Z* test. Specifically, we first computed the *Z*‐statistics for each comparison, *z* = ln(HR)/SE, then to assess the statistical difference between the two *z*‐scores, we used the formula, z_diff = (z1‐z2)/sqrt(2), and calculated the *p*‐value, p_diff = 2*(1‐F[abs(z_diff)]), where F is the cumulative distribution function of the standard normal distribution.

### 4.7 OP50 viability assay

A compound or vehicle was added at the desired concentration (max 0.5% DMSO) to a final volume of 100 μL of liquid LB in a round‐bottom 96‐well plate, 7 wells each per condition. 1 μL of 30 mg/mL OP50 was added to the first experimental well per condition, and 6 1:10 serial dilutions of OP50 were subsequently carried out. Plates were incubated in a shaker at 37°C overnight (18 h). The next day, OP50 was allowed to pellet, and plates were imaged to assess OP50 growth. The quality of the pellet was then manually assigned to “Normal”, “Small”: small circumference, but same thickness, “Sparse”: same circumference, but reduced thickness.

### 4.8 Worm size analysis

The WormLab software (MBF Bioscience) was used to assess the length, width, and area of worms treated with compounds and controls. 20–45 worms were used per group for comparisons. Briefly, the WormLab software uses a set of thresholding methods, and image analysis tools to mask and calculate the length, width, and area of each worm in an image. Results were then statistically compared using a Tukey test with false‐discovery rate (FDR) correction.

### 4.9 Drosophila lifespan assay

Canton S *D. melanogaster* flies were obtained from the Bloomington Drosophila Stock Center (BDSC). 45 male and female flies were sorted upon hatching (virgin flies) and maintained at 25°C on a 12‐h light/dark cycle, at constant 50% humidity. Expired animals were counted by visually assessing lack of movement inside the vial. The compound was mixed into a Cornmeal diet, supplemented with 0.05% ampicillin to prevent bacterial growth.

## AUTHOR CONTRIBUTIONS

G.P. performed all the worm experiments and cowrote the manuscript. J. M. performed data and statistical analysis. C. P. performed the worm and fly experiments. L. S. helped with the worm experiments. S. G. helped prepare the worm and fly experiments. A. O. cosupervised the work, designed the study, and cowrote the manuscript. K. P. cosupervised the work, designed the study, performed data analysis and visualization, and cowrote the manuscript.

## FUNDING INFORMATION

This study was funded by EPITERNA.

## CONFLICT OF INTEREST STATEMENT

None declared.

## Supporting information


Figure S1.

Table S1.

## Data Availability

All data produced in the present work are contained in the manuscript. No additional data available.
