# Supplementary material for: Comprehensive evaluation of lifespan‐extending molecules in C. elegans
Source: Aging Cell. 2025 Jan 24;24(4):e14424. doi: 10.1111/acel.14424 (PMC11984673; doi:10.1111/acel.14424)
Supplement: Supplementary file 1 — Figure S1. [file ACEL-24-e14424-s001.docx]

**Figure S1**

**Figure S2**

**Figure S3**

**Figure S4**
